# Synthesis and catalytic application of magnetic Co–Cu nanowires

**DOI:** 10.3762/bjnano.8.178

**Published:** 2017-08-25

**Authors:** Lijuan Sun, Xiaoyu Li, Zhiqiang Xu, Kenan Xie, Li Liao

**Affiliations:** 1 School of Chemical Engineering, Sichuan University, Chengdu 610065, PR China

**Keywords:** catalyst, Co–Cu nanowires, liquid phase reduction, magnetic materials, metal replacement

## Abstract

A rapid, template-free method was developed to prepare magnetic, bimetallic Co–Cu nanowires via liquid phase reduction and metal replacement under an external magnetic field. The characterization results confirmed that the as-prepared product was bimetallic Co–Cu nanowires with a desirable linear structure. Additionally, the magnetic hysteresis loop showed that the bimetallic Co–Cu nanowires were paramagnetic, which meant they could be easily separated from the reaction mixture. Furthermore, they were applied to the hydrolysis system of ammonia borane as a catalyst for the first time. More importantly, the catalysis results showed that the bimetallic nanowires possessed appealing catalytic performance. Therefore, a rapid and facile synthesis method is introduced which is capable of preparing bimetallic Co–Cu nanowires with great potential for industrial applications.

## Findings

In recent years, many researchers have been devoted to the preparation and characterization of Co–Cu nanowires due to its significant magnetic [1–6] and chemical properties [7]. However, it is worth noting that there are only few papers which report on the application of Co–Cu nanowires.

At present, the primary synthetic method for Co–Cu nanowires is mainly electrodeposition by different templates, as found in the previous literature. For example, many papers have reported Co–Cu nanowires electrodeposited into a porous anodic aluminum oxide template [1–4]. Additionally, L. Gravier et al. [8] prepared Co–Cu multilayered nanowires by electrodeposition in a polymer matrix. However, the electrodeposition method using templates increases the cost of production and the complexity of the synthetic process. Thus, it is necessary to devote attention to the development of a facile method for the preparation of Co–Cu nanowires.

In the present paper, bimetallic Co–Cu nanowires with a highly desirable linear structure were successfully synthesized via a rapid and facile template-free method [9–11] assisted by magnetic fields. Moreover, they were applied to catalyze the hydrolysis of ammonia borane (NH_3_–BH_3_, AB) for the first time. This result lays the foundation for expanding their application to industrial catalysis.

All chemicals were of analytical reagent grade and used without any further purification. CoCl_2_·6H_2_O, EDTA-2Na, CuSO_4_·5H_2_O, PVP, NaOH, N_2_H_4_·H_2_O, H_2_PtCl_6_·6H_2_O and C_2_H_5_OH were purchased from Chengdu Kelong Reagent Co., Ltd (China). H_3_NBH_3_ was supplied from Aladdin (Shanghai, China). Deionized water was used in all aqueous solutions.

The following reactions took place between two parallel neodymium magnets (60 × 30 × 10 mm^3^) separated 150 mm apart. The magnetic intensity inside the reaction solution was approximately 40 mT as measured by a magnetometer. The detailed synthetic process for the Co–Cu nanowires was as follows. First, 0.14 g of CoCl_2_·6H_2_O and 0.22 g of EDTA-2Na were dispersed in 60 mL of deionized water in a 100 mL polytetrafluoroethylene (PTFE) beaker. Then 0.18 g of PVP was added to the reaction solution. The pH value of the solution was adjusted to 14 by adding 2 g of NaOH, and the temperature of solution was kept at 80 °C. Afterwards, 0.1 mL of 80 wt % N_2_H_4_·H_2_O and 0.12 mL of 0.0253 mol·L^−1^ H_2_PtCl_6_·6H_2_O were added to the solution with gentle stirring. Ultimately, the reaction was over after about 5 min, and the product of Co nanowires was collected and rinsed three times with deionized water and ethanol, respectively. Next, 0.3 mL of 80 wt % N_2_H_4_·H_2_O were added to the cobalt nanowires with 60 mL of deionized water in a glass beaker, and heated in a water bath at a stationary temperature of 80 °C. Additionally, 0.015 g of CuSO_4_·5H_2_O (the starting molar ratio was Co/Cu 10:1) was dissolved in the glass beaker with continued stirring. Finally, after about 5 min, the product was separated from the reaction mixture by neodymium magnets. Similarly, the product of bimetallic Co–Cu nanowires was rinsed three times by deionized water and ethanol.

For the catalytic hydrolysis of AB, 10 mL of deionized water and 0.005 g of Co–Cu nanowires were added to a Florence flask and heated at 50 °C in a water bath. Furthermore, 0.023 g of AB was dissolved in the Florence flask with ultrasonic stirring. Ultimately, the volume of hydrogen was monitored by a eudiometer.

The morphology and crystal structure of the products were observed by scanning electron microscopy (SEM, Hitachi S4800), transmission electron microscopy (TEM, FEI Tecnai G20 S-TWIN) and X-ray diffraction (XRD, Rigaku D/max 2200pc, Cu Kα, step size 4 °/min). The composition and amount of the sample was characterized by energy-dispersive X-ray spectroscopy (EDS, Oxford Instruments, X-Max 51-XMX0019). The magnetic performance of the sample was studied by a vibrating sample magnetometer (VSM, Lake Shore Cryotronics Inc., 7400).

**Figure 1:**
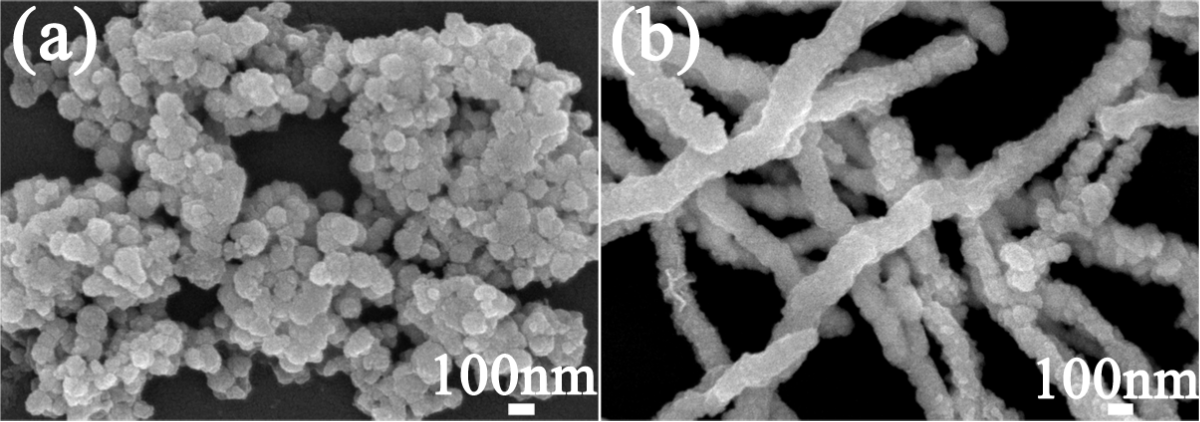
SEM images of bimetallic Co–Cu nanowires (a) without an external magnetic field and (b) with an external magnetic field.

Figure 1 compares the difference between the Co–Cu nanowire synthesis with and without application of an external magnetic field. It was very evident that the linear structure of the Co–Cu nanowires prepared without an external magnetic field was undesirable. Moreover, the Co–Cu nanowires prepared without an external magnetic field resulted in intense aggregation of products, which was found to be a limitation for catalytic applications.

**Figure 2:**
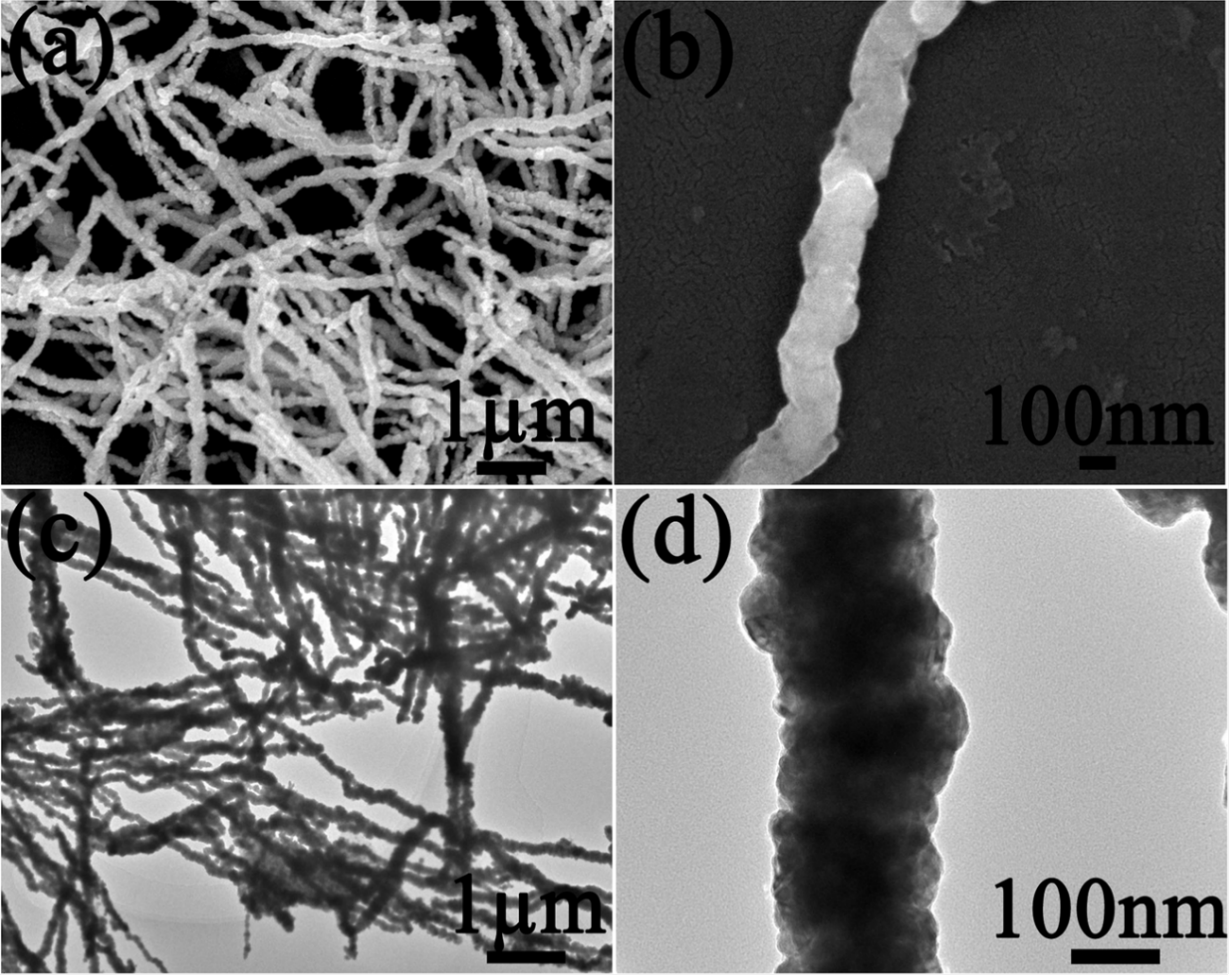
SEM (a) and magnified SEM (b) images as well as TEM (c) and magnified TEM (d) images of bimetallic Co–Cu nanowires at different magnifications.

The SEM and TEM images exhibiting the surface morphology of bimetallic Co–Cu nanowires at different magnification are given in Figure 2. It was obvious that bimetallic Co–Cu nanowires were arranged into long, straight nanowires with smooth surfaces. The average diameter of the nanowires was about 150 nm, which can be observed in Figure 2b,d.

**Figure 3:**
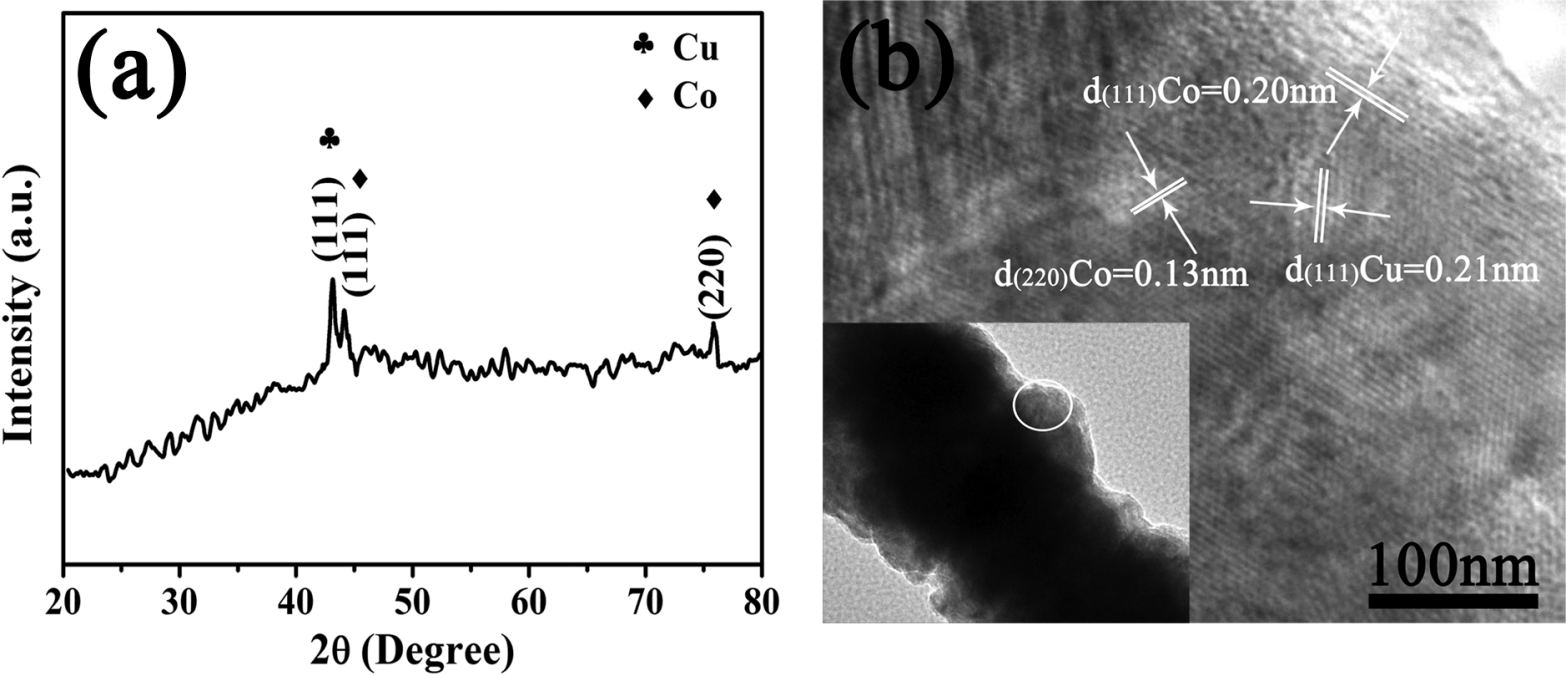
(a) XRD pattern and (b) HRTEM pattern of bimetallic Co–Cu nanowires.

Figure 3 shows XRD and HRTEM patterns of the as-prepared bimetallic Co–Cu nanowires. In Figure 3a, two characteristic peaks of face-centered cubic Co at 2θ values 44.216° and 75.853° corresponded to Miller indices of (111) and (220), respectively. Additionally, the characteristic peak of face-centered cubic Cu is at 2θ = 43.297° and is ascribed to Miller indices of (111). Finally, the XRD spectrum indicated the product was only made up of face-centered cubic Co and Cu without any impurity peaks; therefore, the product was confirmed to be bimetallic Co–Cu nanowires. Furthermore, The HRTEM pattern in Figure 3b showed that the two types of lattice spacings for Co were about 0.20 nm and 0.13 nm, which were in excellent agreement with (111) and (220) lattice planes of face-centered cubic Co, respectively. Moreover, the lattice spacing of Cu was about 0.21 nm, which was consistent with the (111) lattice plane of face-centered cubic Cu. Hence, the components of the bimetallic Co–Cu nanowires were further confirmed through the HRTEM result.

**Figure 4:**
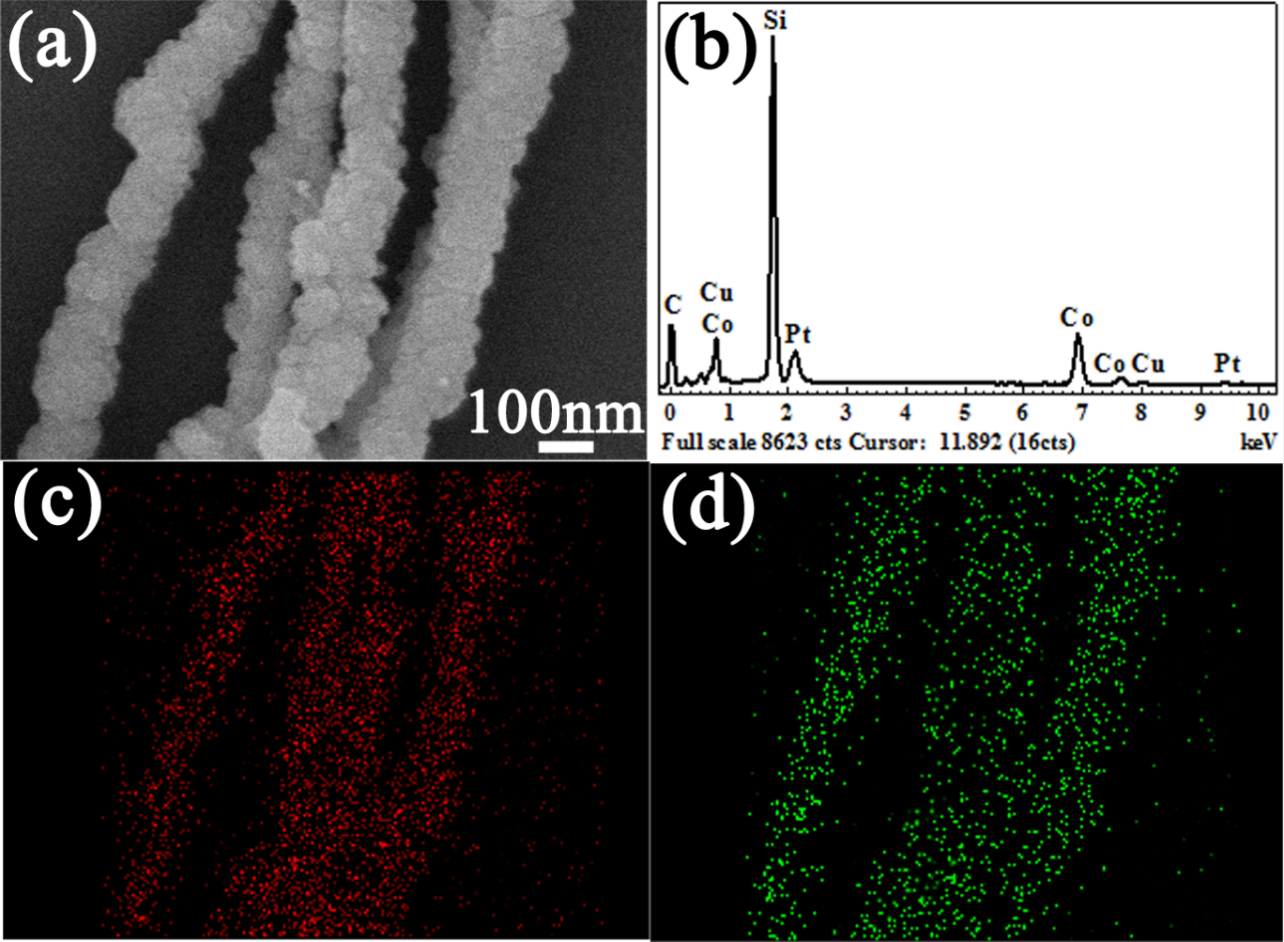
(a) SEM image and corresponding (b) EDS spectra of bimetallic Co–Cu nanowires, and elemental mapping profiles of (c) Co and (d) Cu.

In order to further analyze the composition of the freshly prepared sample, EDS spectra and elemental mapping profiles of as-prepared bimetallic Co–Cu nanowires were taken as exhibited in Figure 4. Figure 4a shows the measurement selected area of the bimetallic Co–Cu nanowires, and Figure 4b shows the detailed elemental spectrum corresponding to the selected area. Figure 4b shows that the as-prepared product contained elemental cobalt as well as elemental copper, which is in accordance with the XRD patterns. Additionally, silicon, carbon and platinum peaks were from the calculation of the elementary composition because they originated from the silicon wafer substrate, PVP and H_2_PtCl_6_·6H_2_O, respectively. Moreover, the elemental mapping profiles (Figure 4c,d) of Co and Cu further proved the presence of Co and Cu as well as their uniform distribution patterns.

**Figure 5:**
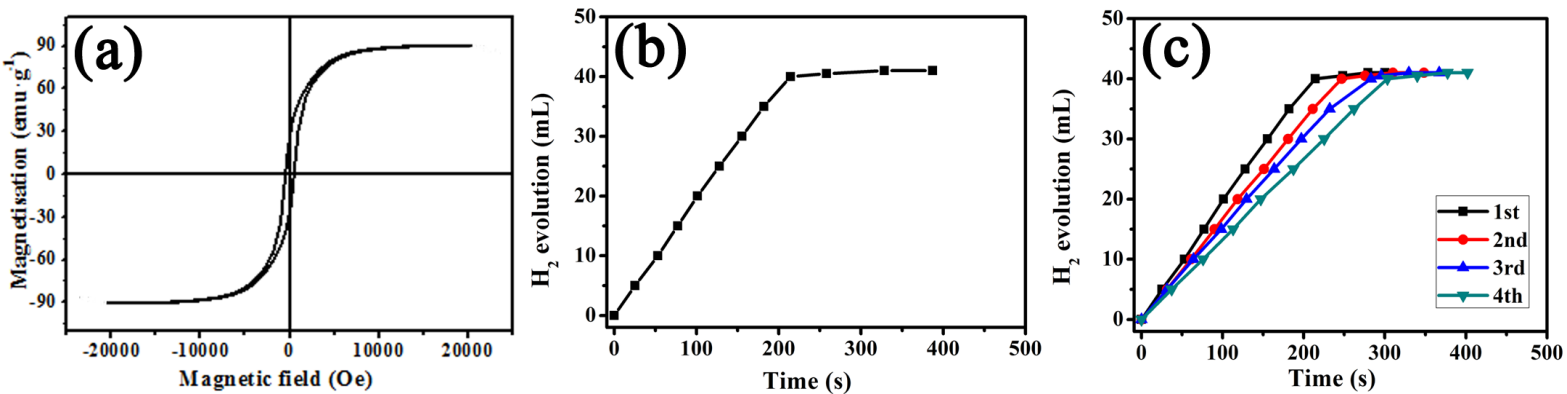
(a) Magnetic hysteresis loop of bimetallic Co–Cu nanowires, (b) time plots of catalytic hydrolysis of AB and (c) hydrogen generation from AB catalyzed by bimetallic Co–Cu nanowires from the 1st to 4th cycles.

The magnetic hysteresis loop of bimetallic Co–Cu nanowires was measured under an applied magnetic field of up to 20,000 Oe at room temperature, and the variation of the magnetization versus magnetic field is exhibited in Figure 5a. The coercivity (*H*_c_), saturation magnetization (*M*_s_) and remnant magnetization (*M*_r_) were 480.30 Oe, 90.47 emu·g^−1^ and 25.50 emu·g^−1^, respectively. The appearance of a hysteresis loop demonstrated that the bimetallic Co–Cu nanowires possessed paramagnetism [12]. Therefore, the bimetallic Co–Cu nanowires could be easily separated from the solution by providing an external magnetic field.

Figure 5b shows the plots of evolution of H_2_ vs time for the hydrolysis of AB using bimetallic Co–Cu nanowires as a catalyst and the activity in terms of turnover frequency (TOF) [13] was calculated to be 6.17 (mol H_2_·min^−1^·(mol Co–Cu)^−1^). Furthermore, bimetallic Co–Cu nanowires could be separated easily from the catalytic system due to their inherent magnetic properties. Therefore, it was convenient to recycle them for the hydrolysis of AB. The recyclability of bimetallic Co–Cu nanowires up to the fourth run for hydrolysis of AB is shown in Figure 5c. Finally, the catalyst retained 70% of its initial catalytic activity in the fourth run.

Table 1 compares the catalytic activity in terms of TOF values of different catalysts used for the hydrolysis of AB. It is clear that Co–Cu nanowires possess enhanced performance as compared to the other catalysts listed in Table 1, and they were much less expensive than these precious metal material based catalysts. Therefore, Co–Cu nanowires show a great promise in the prospect of catalytic systems.

**Table 1:** Catalytic activity of different catalysts used for the hydrolysis of AB.

Catalyst	TOF (mol H_2_·min^−1^·(mol catalyst)^−1^)	Ref.

RGO/Pd	6.25	[14]
Co–Cu NWs	6.17	this work
AuCo alloy	6.0	[15]
PVP-stabilized Ni NPs	4.5	[16]
intrazeolite cobalt(0) NCs	2.4	[17]

## Conclusion

In summary, magnetic bimetallic Co–Cu nanowires were successfully synthesized by a rapid, inexpensive, template-free method under an external magnetic field for the first time. The SEM and TEM results showed that the as-prepared product possessed a highly desirable, linear structure, and the average diameter was about 150 nm. Moreover, the magnetic hysteresis loop indicated that bimetallic Co–Cu nanowires were paramagnetic materials, which meant they could be separated easily from the reaction mixture. Furthermore, the product was successfully applied to the hydrolysis of AB as a catalyst for the first time, and the activity in terms of TOF was calculated to be 6.17 (mol H_2_·min^−1^·(mol Co–Cu)^−1^). Therefore, a rapid and facile synthesis method was developed to prepare bimetallic Co–Cu nanowires with great potential for industrial applications.
